# Functional characterization of a *JAG1* 5'UTR variant in a patient with clinically observed Alagille syndrome

**DOI:** 10.1111/cge.14179

**Published:** 2022-06-27

**Authors:** Nicole Buhl, Eva‐Doreen Pfister, Jens Bohne, Ulrich Baumann, Björn Hartleben, Brigitte Schlegelberger, Thomas Illig, Britta Skawran, Amelie Stalke

**Affiliations:** ^1^ Division of Pediatric Gastroenterology and Hepatology, Department of Pediatric Liver, Kidney and Metabolic Diseases Hannover Medical School Hannover Germany; ^2^ Department of Human Genetics Hannover Medical School Hannover Germany; ^3^ Institute of Virology Hannover Medical School Hannover Germany; ^4^ Institute of Pathology Hannover Medical School Hannover Germany; ^5^ Hannover Unified Biobank Hannover Medical School Hannover Germany

## Abstract

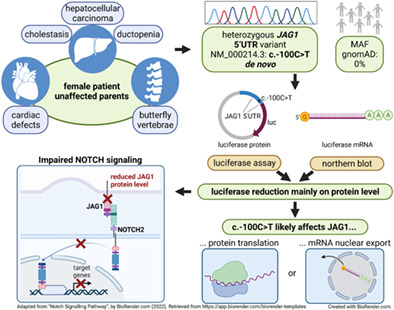

Alagille syndrome (ALGS) is an autosomal dominant disorder characterized by variable abnormalities of liver, heart, face, skeleton, kidneys, vasculature and eyes. It is associated with variants in *JAG1* and *NOTCH2*, encoding for a NOTCH signaling pathway (NSP) ligand and receptor, respectively. Pathogenic *JAG1/NOTCH2* variants commonly affect the coding or splice site region. Here, we functionally characterized the previously by us^1^ detected first *JAG1* 5'UTR variant: NC_000020.10: g.10654278G>A, NM_000214.3: c.−100C>T and classified it as pathogenic (according to the ACMG criteria).

Our female patient with unremarkable family history developed neonatal cholestasis at 4 weeks of age. Liver biopsy demonstrated a ductopenia without significant hepatic fibrosis. Thoracic butterfly vertebrae, mild pulmonary stenosis, a right‐sided aortic arch and mitral regurgitation with a dysplastic valve (surgically corrected at 6 years of age) confirmed ALGS suspicion. After detection of a hepatocellular carcinoma in otherwise biliary cirrhosis at 5 years of age (Figure 1A), she was successfully liver‐transplanted by maternal split donation. The liver function remains normal 17 years later.

**FIGURE 1:**
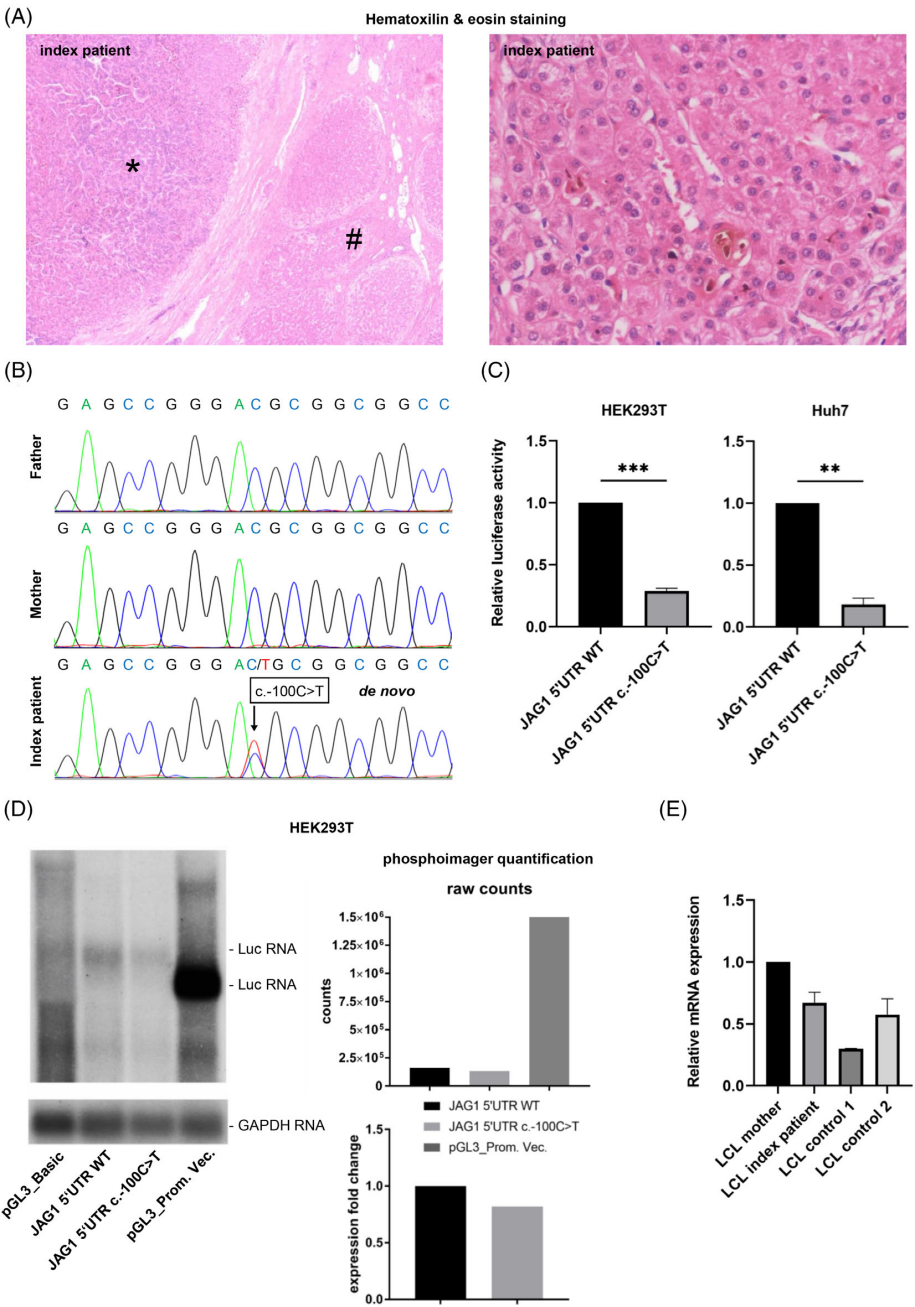
Index patient characteristics and analyses allowing classification of NM_000214.3: c.−100C>T as pathogenic. (A) Paraffin‐embedded liver sections showing hepatocellular carcinoma (*), liver cirrhosis (#) (left), magnified: ductopenia, cholestasis (right). (B) Sanger sequencing. (C) Luciferase assay. Firefly luciferase activity was measured (biological triplicates) after transfection of HEK293T/Huh7 cells with PCR‐amplified (primer: 5'AGTGTGGCTAGCAGCAGCGTCTGCCCAGGTCGC, NheI; 5'AAGTAAAAGCTTACTCGGGACGCCGCCGCTG, HindIII) *JAG1* 5'UTR and promoter sequences (1.121 kb) introduced into pGL3_Basic vector. Normalization to Renilla luciferase activity. Statistics: Paired *t*‐test, **P ≤ 0.01,***P ≤ 0.001. (D) Northern blot. Total RNA of HEK293T after pGL3 transfection as described in C. Controls: pGL3_Basic Vector (Basic) without promoter, pGL3_Promoter Vector (Prom. Vec.) containing an SV40 promoter. E) qRT‐PCR. Relative *JAG1* mRNA expression in lymphoblastoid cell line (LCL) cells (biological duplicates). Quantification: 2^–∆∆Ct^ method using geometric mean of reference genes: *DDX3X, EIF3H, MYCBP2, NAT10, PEA15*. Controls: index patient's mother, further individuals without pathogenic *JAG1* variants (LCL control 1, 2). [Colour figure can be viewed at wileyonlinelibrary.com]

Sanger sequencing of the parents and short tandem repeat analysis confirming maternity and paternity showed that the variant occurred de novo (Figure 1B, criterion PS2). It cannot be found in population data bases/controls (gnomAD; criterion PM2) but in another ALGS patient described by Rajagopalan et al. (criterion PS4_moderate).^2^ Luciferase assays using a vector containing the *JAG1* 5'UTR and promoter regions upstream of the firefly luciferase coding sequence showed significantly decreased luciferase activity for the variant (Figure 1C). This indicates a decreased JAG1 level in vivo and, thus, a pathogenic effect of the variant (criterion PS3_moderate). Variants in the 5'UTR can influence transcription through encoded cis‐regulatory elements or alter translation through a variety of RNA elements. Our northern blot results (Figure 1D) using total RNA suggested that c.−100C>T affects JAG1 expression mainly on the protein level, either due to impaired nuclear mRNA export or impaired translation. *JAG1* expression in lymphoblastoid cell line cells (LCLs) from the patient of Rajagopalan et al. has been decreased also on mRNA level.^2^ However, mRNA analysis of our patient's and her mother's LCL samples and of further controls suggested that the differing mRNA expression might be independent of c.−100C>T and rather due to interindividual variability (Figure 1E). In general, *JAG1* is expressed only weakly in LCLs (https://gtexportal.org/home/gene/JAG1), which do not belong to ALGS‐affected tissues and might not represent the appropriate developmental stage. *JAG1* has been shown to be expressed during human embryogenesis and predominantly in ALGS‐affected tissues.^3^ We cannot expect with certainty that the activity reduction in our luciferase assay occurs exactly to this extent in vivo. However, NSP is highly dose‐sensitive. It signals linearly without amplification, different from many kinase‐mediated pathways.^4^ For c.−100C>T, we detected a 70%–80% reduction in the luciferase protein level in vitro, making a reduced JAG1 protein level and a subsequent biologically relevant reduction of NOTCH2 activation in vivo highly likely. This might favor a strong phenotypic expression as observed in our and the patient described by Rajagopalan et al.^2^ However, variable expressivity and incomplete penetrance, typical for ALGS, can also apply for c.−100C>T. They are likely resulting from NSP's dose sensitivity. Thus, other NSP‐modulating factors may influence signaling. Finally, a mutated *JAG1* 5'UTR may account for ALGS patients without demonstrable *JAG1/NOTCH2* variants. Therefore, *JAG1* genetic testing in patients with suspected ALGS should include the *JAG1* 5'UTR.

## CONFLICT OF INTEREST

Honoraria, lectures, advisory boards, third‐party funding for research projects: Albireo Pharma Inc., Mirum Pharmaceuticals Inc. (UB, EDP) with no role in study design, collection, analysis, interpretation of data, report writing and in decision to report publishing; NB, JB, BH, BS, TI, BSk and AS declare no conflict of interest.

## ETHICS STATEMENT

The study protocol conforms to the ethical guidelines of the Declaration of Helsinki (1964) and has been approved by the Hannover Medical School's ethics committee (#2591–2015, #7656–2018). Patient and parents gave written informed consent.

## FUNDING

This work was supported by the German Research Foundation (DFG) (project #433387263 to AS)

## Data Availability

The data that support the findings of this study are openly available in Global Variome shared LOVD at https://databases.lovd.nl/shared/variants/0000682992#00000084, reference number Variant #0000682992; Individual ID #00307452.
